# Microstructural Evolution and Mechanical Behavior of Thermally Aged Cast Duplex Stainless Steel

**DOI:** 10.3390/ma13245636

**Published:** 2020-12-10

**Authors:** Zhenhua Li, Ying Hu, Tao Chen, Xinyu Wang, Pan Liu, Yonghao Lu

**Affiliations:** 1 State Key Laboratory of Nuclear Power Safety Monitoring Technology and Equipment, China Nuclear Power Engineering Co., Ltd., Shenzhen 518172, China; zhenhuali_ustb@163.com (Z.L.); chent2@cgnpc.com.cn (T.C.); liup@cgnpc.com.cn (P.L.); 2 National Center for Materials Service Safety, University of Science and Technology Beijing, Beijing 100083, China; huying2017@aliyun.com (Y.H.); wangxinyu-010@163.com (X.W.)

**Keywords:** thermal aging, G-phase, small punch test, cast duplex stainless steel, mechanical behavior

## Abstract

The microstructural evolution and mechanical behavior of cast duplex stainless steels (CDSSs) at 400 °C for different thermal aging times were investigated by transmission electron microscope (TEM) and small punch test (SPT). The results showed that the spinodal decomposition in ferrite was the main reason for the decrease in toughness, and G-phase did not play an important role in the embrittlement process. The change of membrane stretching zone (W_m_) played an important role in the SPT load-displacement curve before and after thermal aging. During the deformation process of W_m_ in the SPT, for thermal aging for 10,000 h, some completely curved slip bands were generated inside the ferrite phase, which had no contact with the δ/γ phase interface and belonged to the slip bands produced by the independent deformation of ferrite. The combined effect of the curved slip bands and stress concentration led to the initiation of obvious micro-cracks at the δ/γ phase interface. The micro-cracks propagated along the ferrite phase curved slip bands, and eventually penetrated the entire hardened ferrite phase.

## 1. Introduction

Cast duplex stainless steel (CDSS) is widely used in the primary coolant pipe of a pressurized water reactor (PWR) nuclear power plant because of its excellent comprehensive properties [1,2,3,4,5]. However, CDSS is prone to phase decomposition when it is in the range of 280–500 °C for a long time, resulting in thermal aging brittleness, which reduces the plasticity and toughness of the material and increases the hardness and brittleness, thereby increasing the possibility of a sudden failure of components and affecting the safe operation of nuclear power plants [6,7,8,9,10].

It has been reported that the increase of brittleness in CDSS during thermal aging is mainly due to the generation of the Fe-rich α phase and the Cr-rich α’ phase, and the precipitation of the G-phase for long-term thermal aging [11,12,13,14,15]. Chandra et al. [16] found that the “speckle” feature appeared in ferrite after thermal aging by transmission electron microscope (TEM), which has been confirmed by many studies as the characteristic of spinodal decomposition in ferrite, and the strength of the speckle feature can also represent the degree of spinodal decomposition in ferrite [15,17]. Pareige et al. [18] studied the kinetics of spinodal decomposition and G-phase precipitation in a ferrite of duplex stainless steel after thermal aging treatment by using atom probe tomography (APT) technology, and proved the formation mechanism of G-phase. 

For the CDSS after thermal aging, due to the spinodal decomposition in the ferrite phase and the generation of the G-phase, it is difficult for the hardened ferrite to deform and slip during the deformation process, which reduces the deformation coordination between the austenite and ferrite phases, resulting in the stress concentration at the interface of the austenite and hardened ferrite phases, which ultimately leads to the increase in brittleness and the decrease in mechanical properties. Therefore, it is particularly important to study the mechanical behavior of each phase after thermal aging. At present, many studies have been used to characterize the brittleness and deformation behavior of thermal aging through traditional tensile and impact tests [19,20]. The results show that the appearance of spinodal decomposition and G-phase leads to the hardening of ferrite, and thus increases the brittleness sensitivity of materials. However, due to the large size of the experimental materials required by traditional methods, the utilization rate of materials is not high and it is difficult to test and analyze the equipment components in service, so a more convenient and material-saving method is needed to detect the change in service performance of materials [21].

This study focuses on the microstructural characteristics of CDSS at 400 °C under different thermal aging times, and systematically observes the spinodal decomposition and second phase precipitation behavior in ferrite, revealing and summarizing the evolution of CDSS microstructure before and after the thermal aging process. It then analyzes and discusses the changes in microhardness properties. Moreover, the deformation behavior and fracture mechanism of CDSS before and after thermal aging are characterized and analyzed by means of small punch test (SPT). 

## 2. Material and Experimental Procedure

### 2.1. Test Material

The materials investigated in this study were CDSS Z3CN20-09M, which was obtained from a new component of the primary circuit piping. The chemical composition (wt.%) of the Z3CN20-09M material used in this study was as follows: 20.16 Cr, 8.93 Ni, 0.02 C, 1.07 Si, 1.02 Mn, 0.063 Cu, 0.026 Co, 0.22 Mo, Balance Fe. The test materials were then subjected to a thermal aging treatment at 400 °C, and the thermal aging times were 400, 1000, 5000 and 10,000 h, respectively. According to the Arrhenius equation as shown in Equation (1), the aging effect at 400 °C for 10,000 h was equivalent to 36.4 years at the service temperature (i.e., 290 °C) [22].

(1) $$\frac{t_{1}}{t_{2}}=\exp\left[\frac{Q}{R}\left(\frac{1}{T_{1}}-\frac{1}{T_{2}}\right)\right]$$

where *t_1_* and *t_2_* are the service times at the temperatures *T_1_* and *T_2_*, respectively, *Q* is the Arrhenius activation energy, and *R* is the gas constant.

### 2.2. Test Conditions and Methods

**Figure 1:**
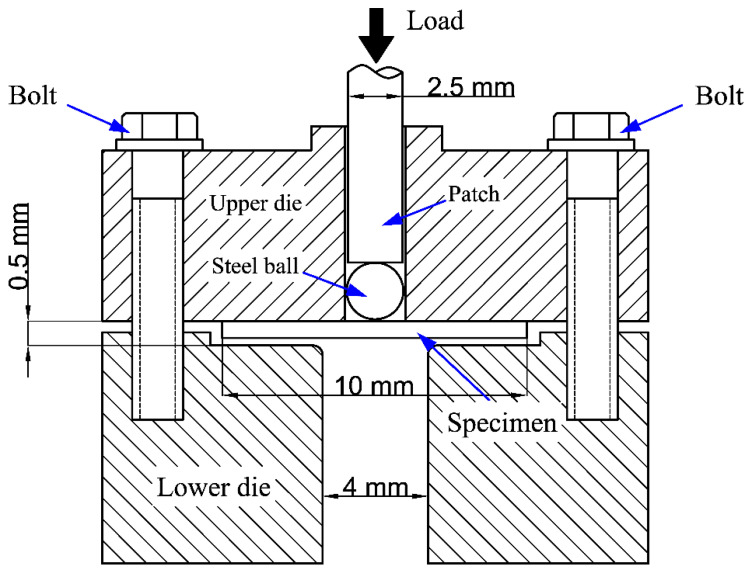
The schematic of the small punch test (SPT) device.

SPT was a semi non-destructive test method. It had been widely considered as an effective test method to obtain the performance data of related materials by using small specimen sizes. Figure 1 is the schematic diagram of the SPT device. The test device was divided into two parts, and the upper and lower parts of the device were fixed by screws. The SPT specimens were discs with a diameter of 10 mm and a thickness of 0.5 ± 0.02 mm. There were three parallel specimens in each thermal aging state. It can be seen that the universal test machine was used to load a 2.5 mm diameter stainless steel punch into the upper part (the load was set to 10 kN), so as to push the specimen deformation to the lower part. The load-displacement curves of each SPT were recorded by the computer of the universal testing machine. During the SPT, the preset loading speed was 0.2 mm/min. All SPTs were performed at room temperature. When the loading dropped to 80% of the maximum load in the test, it was considered that a significant fracture occurred in the SPT specimen, and the material failed. 

The hardness of the ferrite and austenite phases was studied using a Vickers hardness tester under a load of 10 g. Large grain sizes were selected as much as possible, and the indentation should be close to the grain center, so as to avoid the influence of adjacent grains on the measurement results. Eight measurements were made for each test and the hardness value was the average of the eight measurements.

### 2.3. Microstructural Characterization

In order to clearly show the phase interface, the metallographic sample was polished and then electrolytic etched with 10% oxalic acid. The microstructure characteristics under different aging times were observed by optical microscope (OM, Olympus Corporation, Tokyo, Japan). The ferrite contents in the OM images were quantitatively measured with ImageJ software (v.1.8.0, National Institutes of Health, Bethesda, Rockville, MD, USA). The error bars corresponding to the standard deviation were obtained by using the standard deviation function of the origin software. The microstructures before and after thermal aging were analyzed by a high-resolution transmission electron microscope (HRTEM, Tecnai G2 F20, FEI, Portland, OR, USA) operated at 200 KV. After the load-displacements of the SPT were terminated, the deformation and fracture surface morphology of the specimens were observed by scanning electron microscope (SEM, Carl Zeiss, Oberkochen, Germany).

## 3. Results

### 3.1. Microstructural Evolution and Microhardness

**Figure 2:**
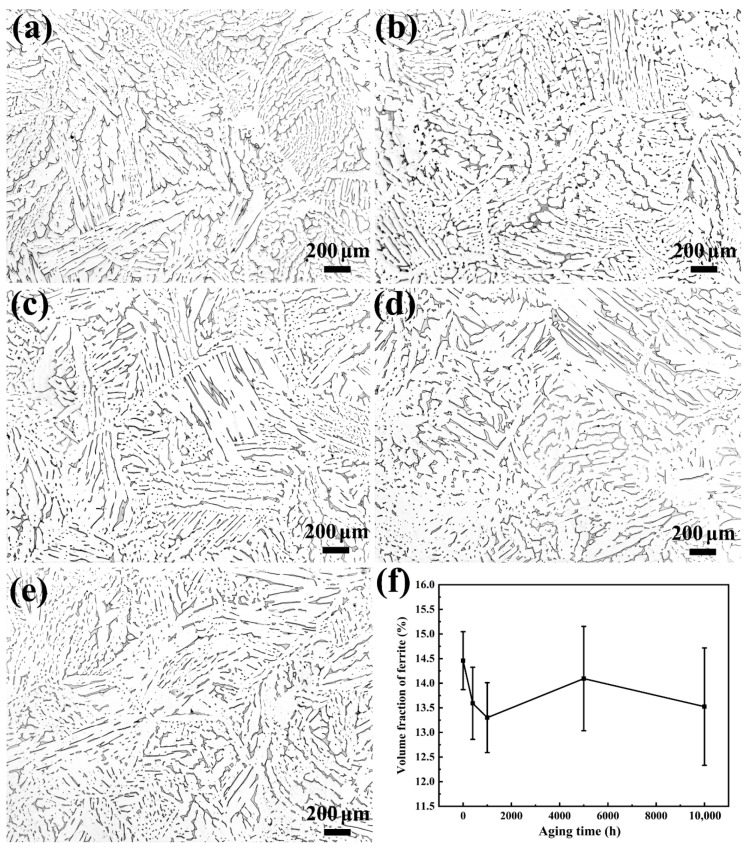
The metallographic microstructure and morphology of CDSS under the condition of thermal aging for (**a**) 0 h, (**b**) 400 h, (**c**) 1000 h, (**d**) 5000 h and (**e**) 10,000 h; (**f**) the corresponding volume fractions of ferrite at different aging times.

Figure 2a–e show the OM microstructure and morphology of the specimens under different thermal aging times. It can be seen that the island- and strip-shaped ferrites (gray δ-phases) are uniformly distributed in the austenite matrix (bright γ-phases), and there is no significant difference in the microstructures of different aging times. It can be seen that the volume fraction of ferrite at different thermal aging times in the stainless steel is about 17% (Figure 2f). With the increase in thermal aging time, the ferrite content in the microstructure does not change significantly. The results show that thermal aging has basically no effect on the macrostructure and morphology of stainless steel.

**Figure 3:**
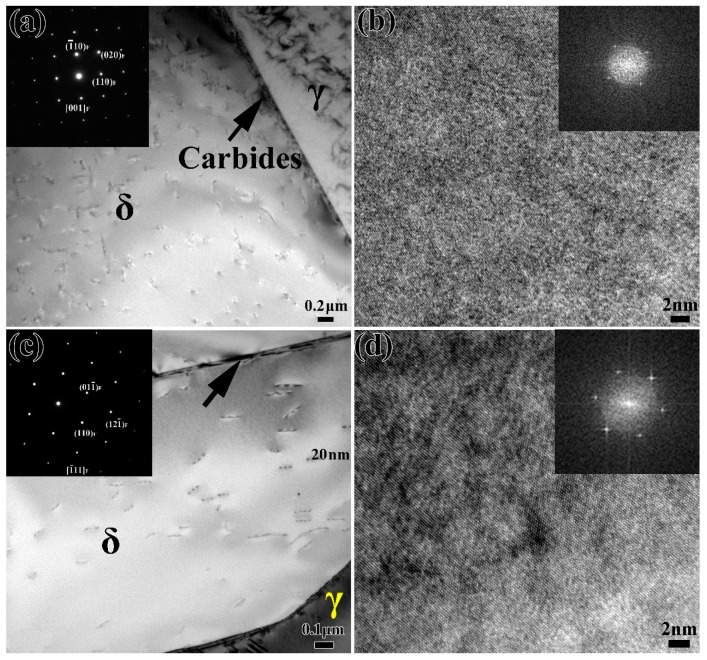
TEM and HRTEM images of stainless steel after aging for (**a**,**b**) 0 h and (**c**,**d**) 1000 h; the corresponding SAED and FFT patterns are inserted.

In order to further study the effect of thermal aging on the ferrite phase of stainless steel, the ferrite phases in the microstructure are observed by TEM and HRTEM. Figure 3a shows the TEM and selected area electron diffraction (SAED) patterns of the unaged specimen (thermal aging for 0 h). There is carbide precipitation along the δ/γ phase interface. Moreover, the corresponding ferrite diffraction pattern shows that there is no second phase in the ferrite. The HRTEM morphology in Figure 3b and the corresponding fast Fourier transform (FFT) patterns further prove that there is no spinodal decomposition or phase precipitation in the unaged ferrite. Figure 3c is the result of the TEM observation of ferrite after 1000 h of thermal aging. It can be seen from the SAED that there is no obvious second phase inside the ferrite. HRTEM and the corresponding FFT patterns in Figure 3d also confirm that no other second phase is precipitated in ferrite. However, it can be found that there are obvious “speckles” in the ferrite, which is a typical feature of spinodal decomposition according to previous reports [13,21,23]. 

**Figure 4:**
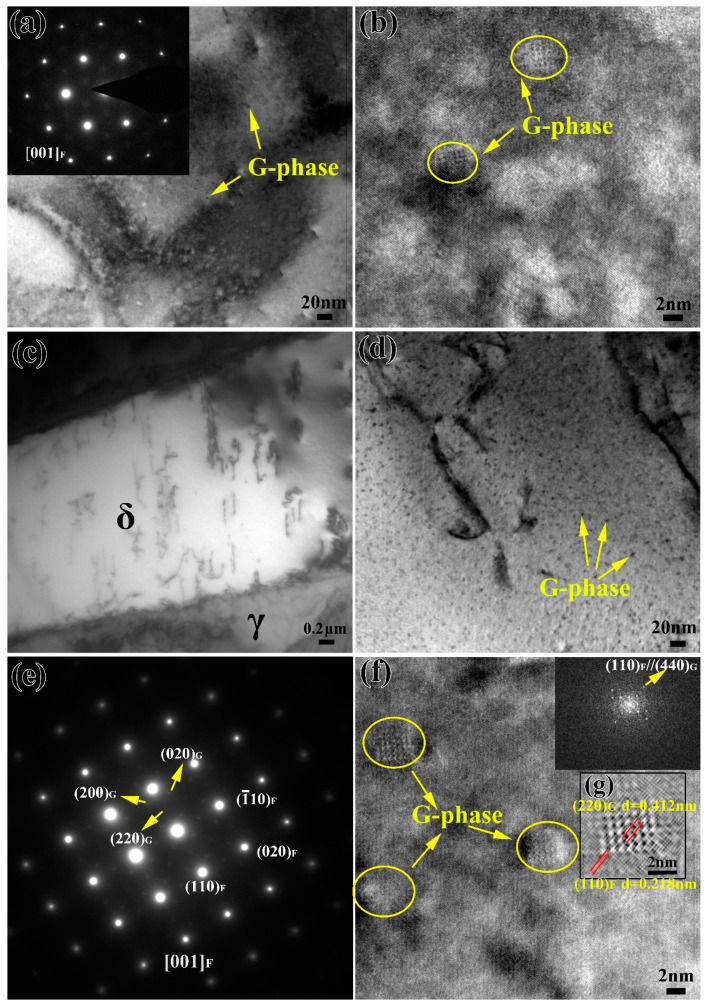
TEM and HRTEM images of stainless steel after aging for (**a**,**b**) 5000 h and (**c**–**g**) 10,000 h; the corresponding SAED, FFT and inverse FFT patterns are inserted.

Figure 4a,b shows the TEM observation results of ferrites aged for 5000 h. It can be seen from Figure 4a that there are obvious black second phase particles in ferrite, and the diffraction spots of the second phase appear in ferrite. The corresponding HRTEM shows the amplified morphology of the precipitates (Figure 4b). Combined with the results of previous literature [24,25], it can be determined that the black precipitates are G-phase. Figure 4c–f shows the results of the TEM observation of the specimen after thermal aging for 10,000 h. The microstructure is shown in Figure 4c, and thermal aging has no significant effect on the macroscopic morphology of ferrite. The high-resolution morphology after thermal aging for 10,000 h is shown in Figure 4d. It can be seen that a large number of second phase particles appear in ferrite, which is significantly greater when compared to the thermal aging for 5000 h. The SAED patterns of ferrite after thermal aging for 10,000 h are shown in Figure 4e. In addition to the diffraction spots of the ferrite phase of the body-centered cubic (BCC) structure, there is another set of G-phases of face-centered cubic (FCC) structures. The latter belongs to the (Fm-3m space group, 225) with the lattice parameter from 1.09 to 1.14 nm, which is exactly four times that of ferrite. Compared with Figure 4a, it can be seen that the diffraction spots of the precipitated phase in ferrite after thermal aging for 10,000 h are more obvious, which indicates that the increase in thermal aging time promotes the growth of the precipitated phase in ferrite. Figure 4f shows the high-resolution morphology of the precipitates in the ferrite matrix and the corresponding FFT patterns. The average size of the precipitates is about 5 nm, which is slightly larger than that after thermal aging for 5000 h. Moreover, the existence of G-phase can be proved by FFT patterns. The corresponding inverse FFT patterns is shown in Figure 4g, and the interplanar distance of the (220) planes of the G-phase is about twice that of the (110) planes of the ferrite. There is a parallelism between planes and directions with the same crystallographic index. The relationships are consistent with those previously reported [19,26] and can be described as:

{100}_F_/ /{001}_G_
{110}_F_/ /{110}_G_
{111}_F_/ /{111}_G_

**Figure 5:**
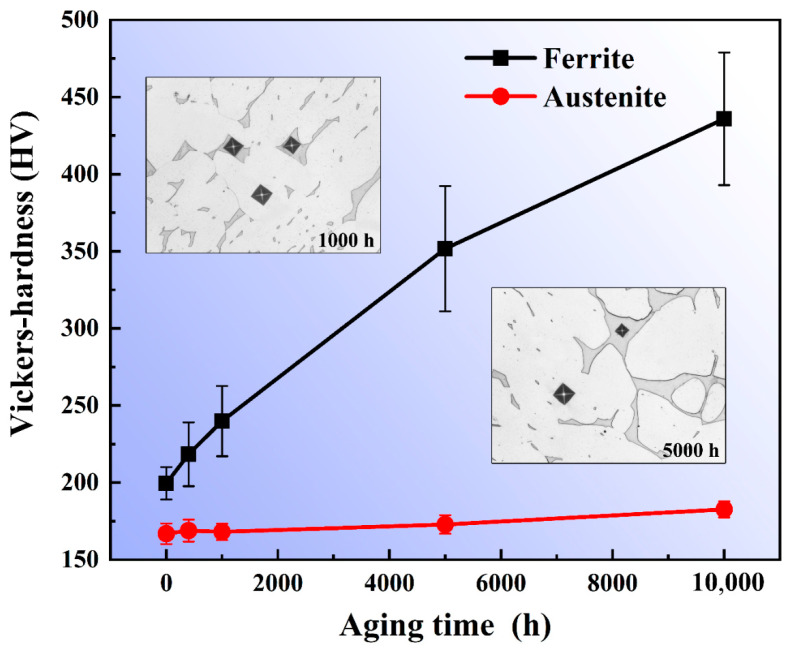
Microhardness of ferrite and austenite phases under different thermal aging time.

The microhardness values of the ferrite and austenite phases under different thermal aging times are shown in Figure 5. It can be seen that the microhardness of the austenite phase has no obvious change with the extension of thermal aging time, but the microhardness of the ferrite phase increases significantly. In the early stage of thermal aging, the increase in microhardness is relatively fast, and the increase rate of microhardness decreases with the extension of the thermal aging time. There is an exponential relationship between the microhardness of the ferrite phase and the aging time. It can also be clearly seen from the microstructure that the area of the square indentation marks appearing in the ferrite aged for 5000 h is significantly reduced compared to that aged for 1000 h under the same loading force, while the austenite has basically no change. The results show that the microhardness change of ferrite during thermal aging is mainly caused by the transformation of the ferrite microstructure.

### 3.2. Small Punch Test

**Figure 6:**
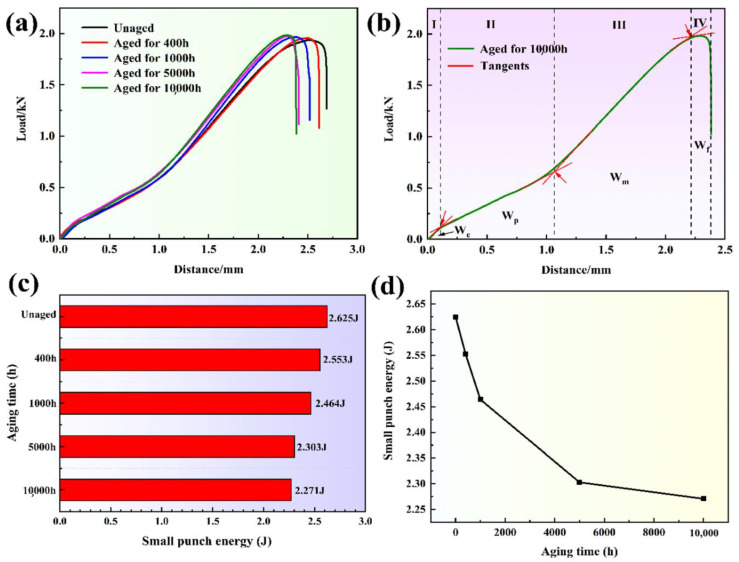
(**a**) The load-displacement curves of the specimens under different thermal aging times, (**b**) the classification diagram of the load-displacement curve area of the unaged small punch specimen, (**c**,**d**) the change chart of small punch energy with thermal aging time.

Figure 6a shows the load-displacement curves of the specimens under different thermal aging times. It can be seen that the load-displacement curves of the specimens under different thermal aging times are obviously different. Under the same displacement, as the thermal aging time increases, the required load gradually increases. In addition, the maximum displacement gradually decreased from 2.64 mm to about 2.38 mm with the increase in thermal aging time, which indicates that the specimen after thermal aging is more likely to produce fracture behavior at a lower displacement, thus leading to failure. The classification diagram of the load-displacement curve area of the unaged small punch specimen is shown in Figure 6b. The load-displacement curve can be divided into four parts according to the change of curve slope: (1) the elastic bending deformation zone (W_e_), in which the elastic deformation occurs; (2) the plastic bending deformation zone (W_p_), in which the plastic deformation occurs; (3) the membrane stretching zone (W_m_), in which the material further plastic deformation occurs and the material undergoes obvious elongation; (4) the instability fracture zone (W_f_), in which cracks appear in the small punch specimen, and the material has failed. We can see the load-displacement curves of the four zones with significantly different curvatures. In addition, the W_p_ and W_f_ are the main components of the load-displacement curve in the SPT. Therefore, the changes in the W_p_ and W_m_ of the small punch specimen, especially the W_m_, dominate the changes in the mechanical properties of the material.

It is believed that the area enclosed by the load-displacement curve and the horizontal and vertical coordinates can be defined as the small punch energy, so the small punch energy results of the specimens under different thermal aging times are shown in Figure 6c. The small punch energy of the unaged specimen is 2.625 J, while with the increases in thermal aging time, the small punch energy gradually decreases to 2.553, 2.464, 2.303 and 2.271 J. During thermal aging for 0–1000 h, the energy of the small punch drops rapidly, and as the thermal aging time increases to 10,000 h, the energy of the small punch gradually decreases (Figure 6d). Among the four parts (Figure 6b), the energy values of W_e_, W_p_ and W_f_ change little with the thermal aging time, but the energy of W_m_ changes obviously, which shows that the deformation behavior of the W_m_ is the main reason for the brittleness of the CDSS.

**Figure 7:**
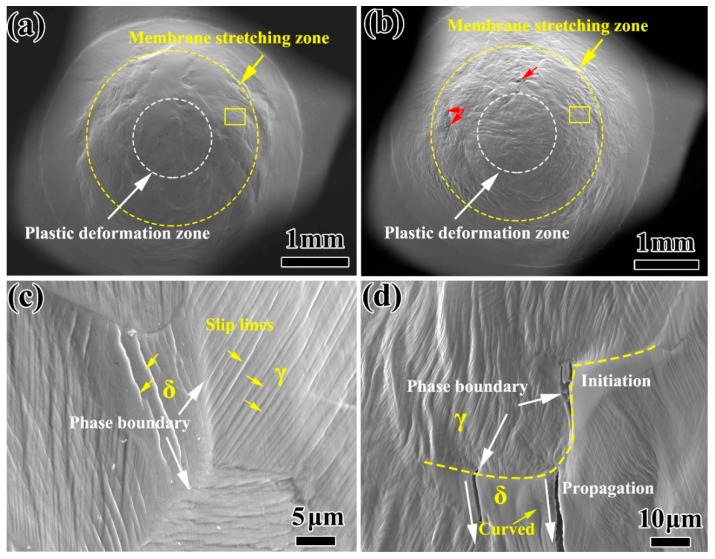
The overall SEM morphology of the small punch specimen after thermal aging for 0 h (**a**) and 10,000 h (**b**) at the same displacement; (**c**,**d**) are the SEM morphology of the corresponding position in the yellow box (**a**,**b**).

In the SPT load-displacement curve, the change of W_m_ plays an important role in the mechanical properties of stainless steel before and after thermal aging. Therefore, the differences in the deformation and fracture behavior in the W_m_ of the specimens with different thermal aging times are analyzed. Figure 7 shows the SEM morphology of the specimens at the same displacement (displacement at 2.2 mm, critical point of unaged W_m_ and W_f_) after thermal aging for 0 h and 10,000 h. It can be seen that the specimen surface mainly includes two zones of W_p_ and W_m_. In addition, the surface of the unaged specimen is still in a good plastic deformation state (Figure 7a), while the surface of the specimen has been wrinkled obviously after thermal aging for 10,000 h, and microcracks appear in W_m_, indicating that the plasticity of the specimen has decreased significantly. The SEM morphology of the W_m_ unaged specimen is shown in Figure 7c. The ferrite and austenite phases show good plasticity and deformation coordination. The deformation in the austenite phase can smoothly drive the ferrite phase to carry on the deformation process. Therefore, the ferrite phase can generate continuous linear slip bands throughout the entire ferrite, which are also known as A2 type slip bands [27]. However, for thermal aging for 10,000 h (Figure 7d), the deformation coordination between austenite and ferrite phases is poor, and the ferrite phase can only pass through independent deformation. Therefore, some completely curved slip bands are generated inside the ferrite phase, which has no contact with the δ/γ phase interface and belongs to the slip bands produced by the independent deformation of ferrite, also known as F1 type slip bands [27]. Austenite and ferrite have different slip direction and deformation on both sides of the phase boundary. Most of the slip lines are blocked by the phase boundary, and gather at the phase boundary and change direction [28]. Microcracks initiate at the δ/γ phase interface and propagate to the ferrite phase. 

**Figure 8:**
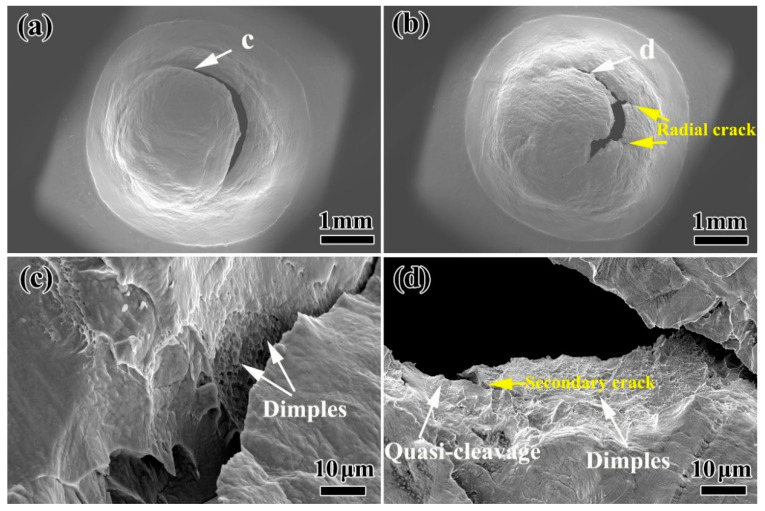
The overall SEM morphology of the small punch specimen after thermal aging for (**a**) 400 h and (**b**) 5000 h when the loading decreases by 80% of the maximum; (**c**,**d**) are the SEM morphologies of the corresponding positions in the white arrows (**a**,**b**).

Figure 8a shows the SEM morphology of the small punch specimen after thermal aging for 400 h. It can be seen that there is a circumferential crack on the surface of the specimen. Figure 8c is the SEM morphology at the white arrow in Figure 8a. The specimen has good plasticity and a large number of dimples on the fracture surface, which is mainly a ductile fracture. Figure 8b shows the surface morphology of the small punch specimen after thermal aging for 5000 h. Compared with the thermal aging of 400 h, the thermal aging of 5000 h has a long circumferential crack and axial cracks on the surface of the specimen. Moreover, the impact depth and range of the specimen are obviously smaller than those of the thermal aging for 400 h, indicating that the absorbed strain energy is less. By observing the fracture morphology at the white arrow (as shown in Figure 8d), it can be seen that the 5000 h aged specimen shows obvious brittleness characteristics. In addition to a few dimples, there are some quasi-cleavage phenomena in the fracture morphology of the specimen, and the edges of some areas were torn. The above results show that with the increase in thermal aging time to 5000 h, the initial ductile fracture of stainless steel gradually changes to the mixed fracture mode of ductile fracture and quasi-cleavage.

## 4. Discussion

### 4.1. Effect of Thermal Aging on Microstructure and Hardness

In the binary alloy phase diagram, there was, generally, nucleation-growth metastable phase decomposition and spinodal decomposition of the unstable phase [29]. Therefore, the thermodynamic conditions of spinodal decomposition were such that a small component fluctuation could continuously reduce the free energy in the system, so that the inside of the ferrite phase could be decomposed spontaneously by uphill diffusion. During low-temperature thermal aging, spinodal decomposition occurred in the ferrite, in which the spinodal was approximately uniformly decomposed into the Fe-rich α phase and Cr-rich α’ phase [14,15], while austenite remains basically unchanged. The spinodal decomposition occurred at the beginning of the thermal aging process, and the degree of decomposition increased with the increase in thermal aging time (Figure 3 and Figure 4). For the formation process of the G-phase, it could be divided into two steps: (1) when α phase and α’ phase were formed by spinodal decomposition, the forming elements (Si, Ni, Mn and Mo, etc.) of G-phase were excluded to the interface; (2) when the chemical composition at the interface reached a certain critical value, G-phase began to nucleate and grow [12,13]. The formation of G-phase in ferrite needed a certain incubation period. For different materials, the incubation period was different. The unified statement was that the obvious G-phase would be precipitated after 3000 h of thermal aging. Moreover, the formation of G-phase was not observed at 1000 h of thermal aging in cast duplex stainless steel (Figure 3), and the obvious precipitation of G-phase was observed at 5000 h of thermal aging (Figure 4). Therefore, it could be inferred that the precipitation of G-phase should occur at 1000–5000 h of thermal aging in cast duplex stainless steel. The transformation of the ferrite microstructure during thermal aging directly led to the hardening of ferrite. The elastic stress and strain field caused by the lattice mismatch between the α and α’ phases generated by spinodal decomposition hinder dislocation movement and plastic deformation, thereby increasing the ferrite hardness [16,30].

### 4.2. Effect of Thermal Aging on Mechanical Behavior

**Figure 9:**
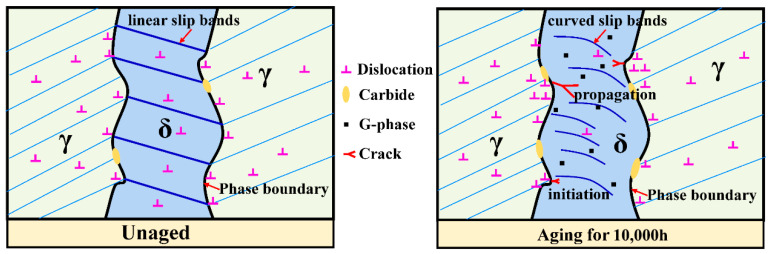
The crack initiation and propagation mechanism of CDSS.

The plastic deformation and rapid decrease in energy in W_m_ (Figure 6) indicated that the plasticity decrease in the CDSS material after thermal aging was the main reason for thermal aging embrittlement. Figure 9 shows the crack initiation and propagation mechanisms of ‘unaged’ and ‘aging for 10,000 h’. Both the ferrite and austenite phases had better plasticity before the thermal aging, and the material as a whole formed continuous and dense linear slip bands, and these slip lines can pass through the ferrite phase easily, so that the fracture morphology also shows uniformly distributed dimples. However, for the material after thermal aging for 10,000 h, the continuous hardening of ferrite resulted in the increase in deformation incongruity between the ferrite and austenite phases and the concentration of stress at the δ/γ phase interface. Meanwhile, a large number of stacking faults in ferrite and the existence of lattice mismatch led to the formation of F1 type slip bands in ferrite phase. The combined effect of the curved slip bands and stress concentration eventually led to the initiation of obvious micro-cracks at the δ/γ phase interface. The micro-cracks propagated along the ferrite phase curved slip band and eventually penetrated the entire hardened ferrite phase. In terms of macroscopic morphology, it led to the appearance of axial cracks and the generation of mixed fracture modes at the fracture (Figure 8).

In addition, the decrease in small punch energy was mainly divided into two stages. During 1000 h of thermal aging, the decreases in small punch energy were relatively rapid. With the increase in thermal aging time to 10,000 h, the energy of small punch decreased slowly (Figure 8). Combined with previous studies on the microstructure change, only the existence of spinodal decomposition was found in the specimen aged for 0–1000 h, which indicated that the rapid decrease in small punch energy after thermal aging for 0–1000 h was caused by spinodal decomposition. However, with the increase in aging time, the G-phase appeared in the ferrite phase, indicating that the small punch energy was slowly reduced by the combined effect of spinodal decomposition and precipitates. The formation of a large number of G-phases during thermal aging would lead to the lack of Ni in the ferrite matrix, which in turn slowed the rate of spinodal decomposition, thereby reducing the embrittlement sensitivity of the material to a certain extent [16].

## 5. Conclusions

The microstructural evolution and microhardness properties of CDSS during thermal aging at 400 °C were investigated. Moreover, the deformation behavior and fracture mechanism of CDSS before and after thermal aging were characterized and analyzed by means of SPT. The major results can be drawn as follows:

(1) The thermal aging time of ferrite in CDSS began to precipitate the G-phase between 1000 and 5000 h, and the growth of G-phase in ferrite was promoted with the increase in aging time;
(2) During the deformation process of W_m_ in the SPT, the unaged ferrite phase could generate continuous linear slip bands throughout the entire ferrite. However, for thermal aging for 10,000 h, some completely curved slip bands were generated inside the ferrite phase, which had no contact with the δ/γ phase interface and belonged to the slip bands produced by the independent deformation of ferrite;
(3) The combined effect of the curved slip bands and stress concentration led to the initiation of obvious micro-cracks at the δ/γ phase interface. The micro-cracks propagated along the ferrite phase curved slip bands and eventually penetrated the entire hardened ferrite phase.
